# The effects of substitution of hospital ward care from medical doctors to physician assistants on non-adherence to guidelines on medication prescribing

**DOI:** 10.1371/journal.pone.0202626

**Published:** 2018-08-23

**Authors:** Jacqueline M. Bos, Marijke J. C. Timmermans, Gerard A. Kalkman, Patricia M. L. A. van den Bemt, Peter A. G. M. De Smet, Michel Wensing, Cornelis Kramers, Miranda G. H. Laurant

**Affiliations:** 1 Department of Clinical Pharmacy, Canisius Wilhelmina Hospital, Nijmegen, the Netherlands; 2 Radboud Institute for Health Sciences, Scientific Center for Quality of Healthcare (IQ healthcare), Radboud University Medical Center, Nijmegen, The Netherlands; 3 Faculty of Health and Social Studies, HAN University of Applied Sciences, Nijmegen, the Netherlands; 4 Department of Hospital Pharmacy, Erasmus University Medical Centre, Rotterdam, the Netherlands; 5 Department of General Practice and Health Services Research, Heidelberg University Hospital, Heidelberg, Germany; 6 Department of Clinical Pharmacology and Toxicology, Radboud University Medical Centre, Nijmegen, the Netherlands; University of California San Diego, UNITED STATES

## Abstract

**Aim:**

This study determined the effect of substitution of inpatient care from medical doctors (MDs) to physician assistants (PAs) on non-adherence to guidelines on medication prescribing.

**Methods:**

A multicenter matched-controlled study was performed comparing wards on which PAs provide medical care in collaboration with MDs (PA/MD model), with wards on which only MDs provide medical care (MD model). A set of 17 quality indicators to measure non-adherence to guidelines on medication prescribing by PAs and MDs was composed by 14 experts in a modified Delphi procedure. The indicators covered different pharmacotherapeutic subjects, such as gastric protection in case of use of NSAID or prevention of obstipation in case of use of opioids. These indicators were expressed in proportions by dividing the number of patients in which the prescriber did not adhere to a guideline, by all patients that were applicable. Multivariable regression analysis was performed in order to adjust for potential confounders.

**Results:**

1021 patients from 17 hospital wards in the ‘PA/MD model’ group and 1286 patients from 17 hospital wards in the ‘MD model’ group were included. Two of the 17 quality indicators showed significantly less non-adherence to guidelines for the PA/MD model; the indicators concerning prescribing gastric protection in case of use of NSAID in combination with corticosteroids (OR 0.42, 95% CI 0.19–0.90) and in case of use of NSAID in patients older than 70 years (OR 0.47, 95% 0.23–0.95). For none of the other quality indicators for prescribing of medication a difference between the MD model and the PA/MD model was found.

**Conclusion:**

This study suggests that the non-adherence to guidelines on medication prescribing on wards with the PA/MD model does not differ from wards with traditional house staffing by MDs only. Further research is needed to determine quality, efficiency and safety of prescribing behavior of PAs.

## Introduction

Hospital care, nowadays, is characterized by a rising prevalence of chronic diseases, ongoing specialization in medical disciplines and increasing dependence on new technologies[1]. To cope with these challenges, many hospitals in different countries have introduced dedicated ward physicians, who are responsible for the delivery and coordination of the daily medical care of hospitalized patients. Their work includes daily ward rounds, performing physical examinations, making decisions regarding necessary tests, treatments and procedures, rendering medical diagnoses and generating and reviewing clinical data [2]. The role of this ward physician has mainly been fulfilled by medical residents or medical specialists[2,3]. The turnover of these ward physicians is traditionally high due to use of recent medical graduates who continue on to do fellowships and the mandatory rotational cycles. In recent years, however, there is an increasing pressure to deliver health care efficiently. Medical procedures are more and more standardized and there are concerns about the continuity, quality and safety of clinical processes [2]. Also, these tasks are now increasingly allocated to physician assistants (PAs) in several countries, among which are the USA, Canada, the UK and the Netherlands. PAs generally do not rotate and constitute a factor of stability in the continually changing medical workforce([4–6].

A PA is a non-physician health care professional licensed to practice medicine in defined domains, in collaboration with MDs but with a substantial degree of professional autonomy [7]. PAs who are employed for medical care for admitted patients usually work in a team comprising both PAs and MDs (i.e. residents, staff physicians or hospitalists). The level of professional autonomy of PAs differs between countries. The scope of practice is determined by law as well as by the competencies of the PAs, the comfort level of the MD with the PA, and the perceived needs in health care delivery. Although there is a worldwide trend of an increase of PAs in the management of hospitalized patients, scientific evidence on the impact of PAs on health care outcomes, quality and safety of care, and costs is limited. Although evidence is scarce, some studies suggest that quality and efficiency of care provided by PAs is similar to that of MDs [8–14].

Prescribing medication is considered a fundamental part of medical ward care practice. Literature shows that a significant part of admitted patients experience one or more adverse drug events (ADEs) during their hospital stay [15–18]. Approximately 50% of these ADEs is potentially preventable. They mainly derive from prescribing errors that lead to potentially preventable morbidity, mortality and costs [15–18]. Research on prescribing errors is mainly focused on prescription of medication by physicians [15–18].

Since January 2012, legislation in Dutch healthcare authorizes PAs to prescribe medication without supervision of a MD [19–20]. Evaluation of this newly acquired authority in 2015 showed that the measure had led to legalization of reserved medical procedures, already performed by PAs. It has created a perspective for PAs to further develop their profession in daily practice [20]. However, scientific evidence on the quality of drug prescribing or adherence to clinical practice guidelines by PAs is hardly available. Published research about quality of drug prescribing by nurses and nurse practitioners (NPs) suggests that this is overall safe. This conclusion should however be interpreted cautiously given the methodological weaknesses in the body of research [19]. Furthermore, the scope of drug prescription by NPs differs from that of PAs, since PAs prescribe more types of drugs to a higher diversity of patients compared to NPs[21].

In this study, we determined the effect of substitution of inpatient care from MDs to PAs on the non-adherence to guidelines on medication prescribing.

### Study aim

The aim of this study was to compare the non-adherence to guidelines on medication prescribing on hospital wards where PAs fulfill the role of ward physician, in collaboration with MDs, to the wards where the role of ward physician is solely fulfilled by MDs.

## Methods

### Study design and setting

This study was conducted as part of a multicenter matched-controlled study comparing wards utilizing a mixed ‘PA/MD model’, on which PAs provide medical care in collaboration with MDs, with wards utilizing a ‘MD model’, on which only MDs provide medical care. This study has been described in detail previously [22].

In short, the study aimed to measure the effects of substitution of inpatient care from MDs to PAs on length of hospital stay, several indicators for quality and safety of inpatient care and patient experiences. 17 wards of the MD model were matched with 17 wards of the PA/MD model based on medical specialty and hospital type (i.e. academic versus non-academic).

Hospital wards were assigned to the PA/MD group if PAs were employed at the wards as substitutes for residents or medical specialists, taking care of inpatient management and daily clinical care. The PAs had to cover at least 51% of the available ward care hours per week during dayshifts (8 a.m. till 6 p.m.) on weekdays and had to have completed a master’s PA degree. Both PAs and residents who provided medical care for admitted patients were supervised by an attending physician of the medical specialty of the ward.

Wards were assigned to the control group if medical care was exclusively provided by MDs. Most of the MDs were residents. The resident is physically present at the department for at least a few hours each weekday, and is the first point of access to medical care. They are supervised by attending medical specialists. In some smaller hospitals, the medical specialists provide all medical care for the admitted patients.

In all hospitals in the Netherlands a computerized physician order entry (CPOE) system is implemented to support prescribing. Hospital pharmacists check medication of all patients on a daily basis with the aid of computer-generated alerts based on a national database (‘G-standard; www.z-index.nl) and with clinical decision support (CDS) systems combining clinical patient data (like renal function and electrolyte levels) with the medication to assess. If necessary, hospital pharmacists warn the prescriber for specific prescribing errors.

### Study population

Patients admitted to 34 different hospital wards across 23 hospitals were included. Terminally ill patients, patients younger than 18 years and patients in daycare (hospital admission of 24 hours or less) were excluded.

### Outcome measures

A set of quality indicators based on pharmacotherapeutic guidelines was composed to measure the quality of prescribing by PAs and MDs.

A set of 17 indicators was composed by means of a consensus procedure. First, provisional indicators were selected from scientific literature by a hospital pharmacist (JB) and an internist-clinical pharmacologist (CK). The selected quality indicators had to relate to medication that has shown to frequently be involved in potentially preventable, clinically relevant, drug-related problems [15–17,23,24]. The indicators should be clearly referenced in a national clinical practice guideline or in the SmPC (Summary of Product Characteristics) of the concerned drug. The indicators had to be part of general knowledge of the prescriber, and the prescriber should be able to perform on these indicators aided by implemented hospital guidelines. Selection was also based on potential relevance for a diversity of medical specialties and available data from the matched-controlled study.

Second, an expert panel of five hospital pharmacists and nine medical specialists (two internist-clinical pharmacologists, one geriatrician-clinical pharmacologist, one nephrologist-clinical pharmacologist, one surgeon and four internists) were approached to participate in a modified Delphi procedure [25]. We asked the expert panel to score the list of provisional indicators on their relevance to determine the quality of drug prescription by physicians on the ward. Scoring was done independently by e-mail. A nine-point Likert scale ranging from 1 (hardly relevant) to 9 (extremely relevant) was used to rate the indicators and also a category ‘could not assess’ was available. In addition, we asked for suggestions for new indicators. In case the suggestions for new indicators were measurable, we included the indicator in a second consensus round. We used a rating scale based on the RAND appropriateness method [26]. Indicators with a median score of at least seven were considered as face valid and relevant and were selected for the final set of indicators. However, in case of too much diversity in scores for one indicator (i.e. at least 30% of the scores as well in the lowest tertile as in the highest tertile), the indicator was not selected[27]. In Table 1 we present the final set of 17 included quality indicators for prescribing. The indicators covered different pharmacotherapeutic subjects, such as gastric protection in case of use of NSAID or salicylates, prevention of obstipation in case of use of opioids, adequate prescribing in case of impaired renal function, adjustment of medication in case of use of iodinated radiocontrast agents, prevention of toxicity of methotrexate and avoidance of certain medication in combination with vitamin K antagonists.

**Table 1:** Indicators to measure adherence to guidelines on medication prescribing.

Quality indicator	Reference indicator	Median score by experts^a^
***Gastric protection***		
1. Check whether a proton pump inhibitor was added in all patients with an ulcer in history and use of an NSAID.	NSAID use and prevention of gastric damage (guideline 2003 CBO)[28]	9
2. Check whether a proton pump inhibitor was added in all patients with an age of older than 70 years and use of an NSAID.	NSAID use and prevention of gastric damage (guideline 2003 CBO)[28]	8
3. Check whether a proton pump inhibitor was added in all patients with use of coumarines in combination with an NSAID.	NSAID use and prevention of gastric damage (guideline 2003 CBO)[28]	9
4. Check whether a proton pump inhibitor was added in all patients with use of corticosteroids in combination with an NSAID.	NSAID use and prevention of gastric damage (guideline 2003 CBO)[28]	7,5
5. Check whether a proton pump inhibitor was added in all patients with an age of older than 80 years and use of salicylates.	Recommendations of the Dutch HARM-Wrestling Task Force [29]	8
***Prevention of obstipation***		
6. All patients with use of an opioid were checked whether a laxative was added. Patients with intestinal stoma were excluded.	Pain (guideline NHG)[30] Diagnostics and treatment of pain[31]	8
***Impaired renal function***		
*Drugs with a contraindication*		
7. All patients with impaired renal function (MDRD<30 ml/min/1.73m^2^) were checked whether an NSAID was avoided.	Dutch national G-standard; SmPC NSAID [32,33]	9
8. All patients with impaired renal function (MDRD<30 ml/min/1.73m^2^) were checked whether nitrofurantoin was avoided.	Dutch national G-standard; SmPC nitrofurantoin [32,33]	7,5
9. All patients with impaired renal function (MDRD<30 ml/min/1.73m^2^) were checked whether dabigatran was avoided.	Dutch national G-standard; SmPC dabigatran [32,33]	8
10. All patients with impaired renal function (MDRD<30 ml/min/1.73m^2^) were checked whether metformin was avoided.	Dutch national G-standard; SmPC metformin[32,33]	8
*Dose adjustment*		
11. All patients with impaired renal function and use of a therapeutic dose of LMWH were checked whether the therapeutic dose of LMWH was adjusted.	Dutch national G-standard; SmPC LMWH([32,33]	8
***Use of iodinated radiocontrast***		
12. All patients, that received iodinated radiocontrast because of imaging diagnostic examination and use of diuretics, were checked whether the diuretic was discontinued on the day of the test.	Precautions for use of iodinated radiocontrast (guideline 2007 NVR)[34]	7
13. All patients, that received iodinated radiocontrast because of imaging diagnostic examination and use of an NSAID, were checked whether the NSAID was discontinued on the day of the test.	Precautions for use of iodinated radiocontrast (guideline 2007 NVR)[34]	8
14. All patients, that received iodinated radiocontrast because of imaging diagnostic examination and a MDRD<60 ml/min/1.73m^2^, were checked whether the metformin was discontinued on the day of the test.	Precautions for use of iodinated radiocontrast (guideline 2007 NVR)[34]	7,5
***Prevention of toxicity***		
15. All patients, that use methotrexate, were checked on concurrent use of folic acid.	Diagnostics and treatment of rheumatoid arthritis (guideline 2009 CBO)[35]	7
***Dosing of vitamin K antagonists***		
16. All patients with use of vitamin K antagonist were checked whether miconazole was avoided.	The art of dosing of vitamin K antagonists (guideline FNT 2016)[36]	8
17. All patients with use of vitamin K antagonist were checked whether cotrimoxazol was avoided. Only for patients with PJP this combination was allowed.	The art of dosing of vitamin K antagonists (guideline FNT 2016)[36]	8

^a^: median score of an expert panel on a nine-point scale (rated 1 to 9), performed to assess the relevance of the indicator in determining the adherence to guidelines on medication prescribing.

Abbrevations: FNT = Federation of Dutch Anticoagulant Services, NSAID = Non steroidal anti-inflammatory drug, MDRD = modification of diet in renal disease, LMWH = Low molecular weight heparin, NHG = Dutch Society of General Practitioners, NVR = Dutch Association of Radiology, PCP = pneumocystis jiroveci pneumonia

The outcome measure of this study is the non-adherence to guidelines on medication prescribing measured by 17 quality indicators. These indicators are expressed in proportions by dividing the number of patients in which the prescriber did not adhere to a guideline, by all patients that were applicable. The MD model served as the reference category.

### Data collection

All required data for the quality indicators were retrospectively derived from patient medical records by trained medical students and researchers. To ensure validity, a random sample of 10% of the patient records per ward was analyzed by a second researcher, who was blinded for the outcome of the initial researcher. In case of an inter-rater agreement of less than 95%, the records of the total sample were reassessed.

### Sample size and data analysis

The sample size was calculated to detect a relative difference of 20% in length of stay (LOS), which is the primary outcome measure of the multicenter study. 34 Wards (17 in each arm) with 100 patients each were calculated to be required [22,37]. This study has been performed as a post-hoc analysis. 17 different quality indicators were used to measure adherence to guidelines on medication prescribing. The numbers of cases for e.g. impaired renal function, gastric protection, or use of iodinated radiocontrast in the study population was not known in advance. Therefore, for the present study, no sample size calculation was performed.

Baseline characteristics of the study population are presented as mean and standard deviations (mean ± SD) for continuous variables, and proportion (%) for categorical variables. Quality indicators were expressed as proportions by dividing the number of patients in whom the prescriber did not adhere to a guideline, by the number of patients to which the guideline applied. To compare differences on the selected indicators between the PA/MD model and the MD model, logistic regression analyses were performed. Multivariable models were constructed to correct for relevant differences between the groups at baseline. Relevant variables were chosen on basis of availability, relevance for the outcome measures based on literature and feasibility concerning numbers of cases. The study has been matched for the variables hospital type and medical specialty. Other covariables were included in the final model only if they modified the regression coefficient of the central determinant by more than 10%, regardless of statistical significance of effects. Associations were expressed as odds ratios with 95% confidence interval. In all analyses, two-tailed p-values of 0.05 or lower were considered statistically significant. Analyses were performed with SPSS statistics version 24 (IBM Software, USA).

### Ethical considerations

Ethical approval was sought from the Research Ethics Committee of the Radboud University Nijmegen Medical Centre (registration number: 2012/306); the committee judged that ethical approval was not required under Dutch National Law. All data were handled strictly confidential and written informed consent was obtained from all patients.

## Results

1021 Patients from 17 hospital wards were included in the ‘PA/MD model’ and 1286 patients from 17 hospital wards were included in the ‘MD model’. The main characteristics of the patients are summarized in Table 2. Most characteristics were well balanced between the groups. Less patients in the PA/MD model group were admitted electively in comparison with the MD model. (41% versus 56%).

**Table 2:** Baseline characteristics of patients.

Baseline characteristic	PA/MD model (n = 1021)	MD model (n = 1286)
Medical specialty *n(%)*		
Surgery	601 (59%)	696 (54%)
Gastroenterology	102 (10%)	181 (14%)
Pulmonology	91 (9%)	107 (8%)
Cardiology	101 (10%)	124 (10%)
Orthopaedics	103 (10%)	100 (8%)
ENT, head and neck oncology surgery	23 (2%)	78 (6%)
Hospital type *n(%)*		
Teaching	552 (54%)	709 (55%)
Academic	23 (2%)	78 (6%)
Non-academic	529 (52%)	631 (49%)
Non-teaching	469 (46%)	577 (45%)
Cover of PA in ward care, *median (%)*	68 (IQR 48–77)	0
Gender, male *n(%)*	524 (53%)	682 (54%)
Age, years *mean ± SD*	64 ± 16	63 ± 15
Primary diagnoses *n(%)*		
Digestive system	204 (20%)	247 (19%)
Circulatory system	158 (16%)	274 (22%)
Neoplasms	108 (11%)	195 (15%)
Musculoskeletal system and connective tissue	120 (12%)	119 (9%)
Injury and poisoning	135 (13%)	80 (6%)
Infectious diseases	59 (6%)	81 (6%)
Respiratory system	51 (5%)	75 (6%)
Charlson index for co-morbidity score *mean ± SD (% with score ≥1)*	1.1 *±* 1.8 (43%)	1.1 *±* 1.8 (44%)
Type of admission *n(%)*		
Elective	402 (41%)	687 (56%)
Acute	588 (59%)	547 (44%)

Note: The overall population was used in this non-adherence study. Numbers may not add up to the total because of missing values.

Table 3 shows the results of the quality indicators expressed as the proportion of patients in which the prescriber in the ‘PA/MD model’ group and in the ‘MD model’ group did not adhere to a pharmacotherapeutic guideline and the matching odds ratios with 95% confidence interval.

**Table 3:** Non-adherence to pharmacotherapeutic guidelines, based on the selected quality indicators.

Quality indicator	PA/MD model **	MD model**	Adjusted Odds ratio *	
			OR	95% CI
***Gastric protection***				
1. All patients with an ulcer in history and use of an NSAID were checked whether a proton pump inhibitor was added.	0/6 (0%)	1/11 (9.1%)	NA	
2. All patients with an age of older than 70 years and use of an NSAID were checked whether a proton pump inhibitor was added.	18/113 (15.9%)	25/100 (25%)	0.47	0.23–0.95 (p = 0.037)
3. All patients with use of coumarines in combination with an NSAID were checked whether a proton pump inhibitor was added.	6/27 (22.2%)	6/21 (28.6%)	0.66	0.18–2.48
4. All patients with use of corticosteroids in combination with an NSAID were checked whether a proton pump inhibitor was added.	9/58 (15.5%)	81/248 (32.7%)	0.42	0.19–0.90 (p = 0.012)
5. All patients with an age of older than 70 years and use of salicylates were checked whether a proton pump inhibitor was added.	13/51 (25.5%)	11/50 (22%)	1.41	0.53–3.74
***Prevention of obstipation***				
6. All patients with use of an opioid were checked whether a laxative was added. Patients with intestinal stoma were excluded.	463/606 (76.4%)	590/785 (75.2%)	1.13	0.87–1.46
***Impaired renal function***				
*Drugs with a contraindication*				
7. All patients with impaired renal function (MDRD<30 ml/min/1.73m^2^) were checked whether an NSAID was avoided.	3/24 (12.5%)	0/22 (0%)	NA	
8. All patients with impaired renal function (MDRD<30 ml/min/1.73m^2^) were checked whether nitrofurantoin was avoided.	1/24 (4.2%)	2/22 (9.1%)	0.41	0.03–4.96
9. All patients with impaired renal function (MDRD<30 ml/min/1.73m^2^) were checked whether dabigatran was avoided.	0/24 (0%)	1/22 (4.5%)	NA	
10. All patients with impaired renal function (MDRD<30 ml/min/1.73m^2^) whether checked if metformin was avoided.	7/24 (29.2%)	3/22 (13.6%)	2.58	0.57–11.62
*Dose adjustment*				
11. All patients with impaired renal function and use of a therapeutic dose of LMWH were checked whether the therapeutic dose of LMWH was adjusted.	0/2 (0%)	0/5 (0%)	NA	
***Use of iodinated radiocontrast***				
12. All patients, that received iodinated radiocontrast because of imaging diagnostic examination and use of diuretics, were checked whether the diuretic was discontinued on the day of the test.	48/67 (71.6%)	51/68 (75%)	0.80	0.36–1.78
13. All patients, that received iodinated radiocontrast because of imaging diagnostic examination and use of NSAID, were checked whether the NSAID was discontinued on the day of the test.	44/70 (62.9%)	37/60 (61.7%)	1.02	0.49–2.12
14. All patients, that received iodinated radiocontrast because of imaging diagnostic examination and a MDRD<60 ml/min/1.73m^2^, were checked whether the metformin was discontinued on the day of the test.	2/2 (100%)	4/9 (44.4%)	NA	
***Prevention of toxicity***				
15. All patients, that used methotrexate, were checked on concurrent use of folic acid.	0/8 (0%)	0/12 (0%)	NA	
***Dosing of vitamin K antagonists***				
16. All patients with use of vitamin K antagonist were checked whether miconazole was avoided.	1/124 (0.8%)	1/153 (0.7%)	1.28	0.08–20.71
17. All patients with use of vitamin K antagonist were checked whether cotrimoxazol was avoided. Only for patients with PJP this combination was allowed.	4/124 (3.2%)	0/153 (0%)	NA	

NA = not applicable because of limited number of cases

* Adjusted for type of admission (elective or acute)

** Quality indicators are expressed in proportions by dividing the number of patients in which the prescriber did *not* adhere to a guideline, by all patients that were applicable. The MD model served as the reference category.

Two of the 17 quality indicators showed significantly less non-adherence for the PA/MD model. These were the indicators concerning prescribing gastric protection in case of use of NSAID in combination with corticosteroids (OR 0.42, 95% CI 0.19–0.90) and in case of use of NSAID in patients older than 70 years.

In none of the other quality indicators for non-adherence to guidelines on medication prescribing a difference between the MD model and the PA/MD mixed model was found.

## Discussion

In this study we aimed to determine the effect of substitution of inpatient care from MDs to PAs on non-adherence to guidelines on medication prescribing on the ward. Indicators of prescribing gastric protection in case of use of NSAID by patients with an age over 70 years or in combination with use of corticosteroids showed less non-adherence in the ‘PA/MD model’ group than in ‘MD model’ group. None of the other quality indicators showed a significant association between the involvement of PAs and the quality of prescribing. Adherence to recommendations varied across indicators, but tended to be low overall. Although we have to interpret the results cautiously because of the relatively small sample size for several quality indicators, this study suggests that the non-adherence to guidelines on medication prescribing on wards on which PAs provide medical care in collaboration with MDs (PA/MD model), does not differ from wards on which only MDs provide medical care (MD model).

In recent years the PA has been increasingly introduced in the hospital. A PA is a non-physician health care professional licensed to practice medicine in defined domains and trained to do tasks that were formerly performed by physicians only. The primary motive for employing a PA in Dutch health care is to increase continuity and quality of care [38]. The level of professional autonomy of PAs differs between countries. From the introduction of the PA there has been debate about prescribing by PAs[20]. It has been suggested that only physicians have the capability to prescribe medication and that only physicians should be allowed to do so. Prescribing is viewed as a very complex, risky clinical task. Many studies have shown that prescription errors made by physicians lead to preventable adverse events in hospitals [15–18]. Nowadays, although with different levels of autonomy, in most countries PAs have been authorized to prescribe a limited list of medication, based on the specific training of the PA. Until now, there have been no studies to evaluate prescribing of PAs compared to MDs.

It has been shown that continuity of care is higher on wards with PAs, and that PAs often have more years of experience on the ward than residents do. As a consequence, PAs might be more familiar with prevailing clinical practice protocols and pharmacotherapeutic guidelines [39]. This could implicate that PAs are more capable, dedicated and motivated to follow guidelines. On the other hand, because of a lower degree of autonomy of the PA, it may also be more difficult for a PA to deviate from guidelines, when this is needed in certain circumstances.

We found that the PA/MD model performed better on the quality indicators concerning gastric protection in case of NSAID use in combination with another risk factor. Van den Bemt et al. found that the proportion of admissions in which MDs were not compliant with guidelines on gastric protection in case of use of NSAID in hospitalized surgical patients was 46.6%[40]. In our study we found a better performance in both models, but a significant better result in case of the PA/MD model. For this specific guideline, this could confirm the hypothesis that PAs are more dedicated to follow the guideline.

For the indicator that measures prevention of obstipation in case of use of opioids we found no difference and also a poor adherence to the guideline (non-adherence 76.4% for the PA/MD model and 75.2% for the MD model). We excluded patients with intestinal stoma, but for this guideline there are more situations in daily practice in which a prescriber could deviate intentionally from this guideline. It is possible that better compliance by PAs is masked by a higher percentage of just deviation from guidelines by MDs because of certain patient circumstances. Detailed clinical data necessary to assess justified deviation of the guideline was not available in this study.

We found that a diuretic, NSAID, or metformin in case of renal failure, was often not discontinued when patients received iodinated radiocontrast because of imaging diagnostic examination (68.3% for the PA/MD model and 70.5% for the MD model). Schilp et al. studied adherence to the guideline concerning identification and hydration of high-risk patients for radiocontrast-induced nephropathy in different hospitals and also found that only two third of the high-risk patients were hydrated before contrast administration, but they did not measure discontinuation of drugs according to the guideline [41].

Overall, adherence to guidelines varied across the indicators we measured, but tended to be low. This is in line with earlier research on pharmacotherapeutic guideline adherence [40–42]. Different variables, such as organizational, guideline or patient factors but also health professional factors, that are associated with poor guideline adherence, have been described [43]. Our study suggests that a poor guideline adherence cannot be explained by a difference in prescribing by PAs and MDs.

This study has some methodological strengths and limitations. A major strength of this study is the multicenter design. 15 wards in teaching hospitals and 19 wards in non-teaching hospitals were included. This is approximately in proportion with the general situation in the Netherlands (36 teaching hospitals and 60 nonteaching hospitals). Most of the wards were of a surgical (sub)specialty. We know that, in the Netherlands, most of the PAs are employed at a surgical department.

Another strength of the study is the broad set of indicators that measures non-adherence to guidelines and covers different pharmacotherapeutic subjects. Moreover, this set was composed by a consensus procedure in which 14 medical experts participated in a modified Delphi procedure to select a final set of representative indicators, that are considered clinically relevant for inpatient care.

One of the limitations is the non-randomized design of the study, which implies an increased risk of confounding. We took this into account by performing a multivariable analysis. However, we cannot exclude that there are small local differences like the organization of ward care (care by resident or specialist, arrangement of supervision, levels of experience), policies on the medication process, implementation of CPOE based clinical decision support and pharmacist care and patient case-mix, that are not accounted for in the multivariable analysis. Another limitation of this study is the relatively small sample size for several quality indicators we measured. The primary study was not powered to detect effects on prescribing indicators. Results of the statistical analyses should therefore be interpreted with caution.

The study compared wards with a PA/MD model to wards with traditional house staffing by MDs only. This implicates that prescribing of medication on the wards with the PA/MD model was not only performed by PAs. It is more likely that we measured a team performance than the performance of the PA alone. However, PAs covered most of the available ward care hours per week during dayshifts (68% (IQR 48–77%))[39]. Moreover, measurement on the ward with several medical residents and medical specialists can also be interpreted as team performance.

The PA model has become a global strategy to augment medical service delivery and the concept has spread to more than a dozen countries spanning 4 continents [44]. Although the role of the PA in these countries differs, it is important to obtain more evidence on quality, safety or efficiency of prescribing by PAs to be sure that the complex task of prescribing can be performed by these medically trained professionals. Dedicated research should be designed and performed with measures as guideline adherence, adverse drug events and prescribing errors, linked to relevant clinical patient outcome measures.

## Conclusions

This study suggests that the non-adherence to guidelines on medication prescribing on wards with the PA/MD model does not differ from wards with traditional house staffing by MDs only. Further research is needed to determine quality, efficiency and safety of prescribing behavior of PAs.
